# Ca^2+^-Sensor Proteins in the Autophagic and Endocytic Traffic

**DOI:** 10.2174/13892037112139990033

**Published:** 2013-03

**Authors:** Ghita Ghislat, Erwin Knecht

**Affiliations:** Laboratorio de Biología Celular, Centro de Investigación Príncipe Felipe, C/ Eduardo Primo Yúfera 3, Valencia 46012, Spain and CIBERER, Valencia, Spain

**Keywords:** Autophagy, calcium, endocytosis, lysosomes, membrane fusion.

## Abstract

Autophagy and endocytosis are two evolutionarily conserved catabolic processes that comprise vesicle trafficking events for the clearance of the sequestered intracellular and extracellular cargo. Both start differently but end in the same compartment, the lysosome. Mounting evidences from the last years have established the involvement of proteins sensitive to intracellular Ca^2+^ in the control of the early autophagic steps and in the traffic of autophagic, endocytic and lysosomal vesicles. However, this knowledge is based on dispersed outcomes that do not set up a consensus model of the Ca^2+^-dependent control of autophagy and endocytosis. Here, we will provide a critical synopsis of insights from the last decade on the involvement of Ca^2+^-sensor proteins in the activation of autophagy and in fusion events of endocytic vesicles, autophagosomes and lysosomes.

## INTRODUCTION

Lysosomes are ubiquitous organelles that degrade material sequestered by two main dynamic processes: autophagy and endocytosis. Both processes comprise a complex traffic of vesicles that finally ends with the clearance of their contents by the lysosomal acid hydrolases.

**Fig. (1):**
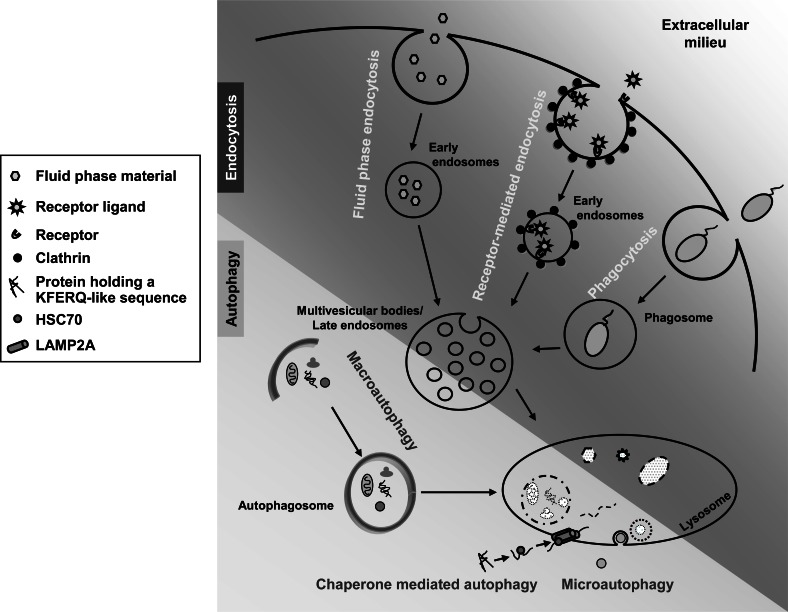
Main endocytic and autophagic pathways. Upper part depicts from left to right: i) fluid phase endocytic uptake of extracellular fluid containing small molecules; ii) receptor-mediated endocytic uptake of specific ligands, generally within clathrin-coated vesicles; and iii) phagocytic uptake of solid particles such as bacteria. Lower part represents from left to right: i) macroautophagy of cytosolic components including organelles; ii) chaperone mediated autophagy of proteins harboring KFERQ-related sequences; iii) microautophagy of cytosolic material. See text for further details.

Autophagy is an important pathway responsible for the turnover of intracellular macromolecules and even whole organelles [1]. At least three different forms of autophagy coexist in the cell (Fig. **1**): microautophagy, chaperone-mediated autophagy and macroautophagy. Microautophagy involves the internalization of cytosolic components by various modifications of the lysosomal membrane [2]. It has been mainly characterized in yeast and it is still poorly understood in eukaryotic cells. Chaperone-mediated autophagy is a more selective form of autophagy by which specific amino acid motifs in cytosolic proteins (KFERQ-like) are recognized by a chaperone (HSC70) that binds to isoform A of lysosome-associated membrane protein type 2 (LAMP2A). This allows, with the help of other chaperones at the lysosome, such as HSP90 and the lysosomal isoform of HSC70, the unfolding and subsequent translocation of the specific substrate proteins into the lysosomal lumen [3]. Finally, macroautophagy is the most prominent and best studied of these three forms and hence it will be simply called autophagy. It starts with the formation of a cup-shaped vesicle, called phagophore, whose origin is still a matter of conjecture, that engulfs cytoplasmic material and closes, thus generating a double membrane vacuole, the autophagosome [4]. Several compartments, including mitochondria [5, 6], plasma membrane [7], Golgi complex [8] and endosomes [9], appear to contribute proteins and lipids to the phagophore [10], but the most accepted origin of this structrue is the endoplasmic reticulum (ER) [11-13]. Once formed, the autophagosome undergoes a maturation process by fusing with late endosomes/lysosomes to acquire proteolytic competence [14]. Analysis of autophagy in yeast led to the identification of a series of autophagy-related genes (*ATG*s), most of them essential for autophagosome formation and whose mammalian homologues are well identified [15]. Many reviews have already discussed the functions of these genes (e.g. [1, 15, 16]), and here we will only provide a brief summary of those mentioned in the text. They include UNC-51 Like Kinase (ULK1) (whose yeast homologue is ATG1), ATG13, FIP200 (ATG17) and ATG101, all of which form a complex involved in the initiation of the phagophore, and WIPI1 (ATG18), which is involved in the nucleation of the autophagosomal membrane. In addition, its elongation is controlled by two complexes. The first is formed by the ATG7-mediated binding of ATG12 and ATG5, which later oligomerize with ATG16L (ATG16). The second is formed by Beclin 1 (ATG6), phosphatidylinositol 3-kinase class III (VPS34), p150 (VPS15) and ATG14L (ATG14). Beclin 1 is a tumor suppressor that under nutrient rich conditions is bound to protein B-cell lymphoma/leukemia 2 (Bcl-2). Under starvation, JNK1 phosphorylates Bcl-2, from which Beclin 1 dissociates and interacts with the above mentioned second complex involved in the elongation of the autophagosomal membrane. Other Beclin 1 partners appear to inhibit, such as Bcl-XL, or to activate, such as Activating molecule in Beclin 1-regulated autophagy (Ambra), autophagosome formation and others, such as Bif1 and Ultraviolet irradiation resistance-associated gene, VPS38 (UVRAG), induce the fusion of autophagosomes with lysosomes. Finally, we should also mention here LC3 (ATG8). Its cytosolic form (LC3-I) can covalently bind to phosphatidylethanolamine under a series of reactions catalyzed by ATG4, ATG7 and ATG3, forming LC3-II that associates to the autophagosomal membrane.

Endocytosis is the process whereby extracellular and plasma membrane materials are internalized and transported to lysosomes by vesicles [17]. During this endocytic traffic, early endosomes undergo maturation and budding/scission events, thereby generating larger and more acidic multivesicular bodies/late endosomes, which are subsequently delivered to lysosomes for the final degradation of the endocytosed cargo (see Fig. **1**). One of the best-characterized forms of endocytosis is receptor-mediated endocytosis, responsible for the selective internalization of specific ligands recognized by their receptors at the cell surface [17]. Phagocytosis and fluid phase endocytosis are other forms of endocytosis in which structures and molecules of variable size are engulfed by the cell [18]. Different proteins are involved in all these endocytic processes that together coordinate the specific and non-specific uptake of extracellular material into the cell and their subsequent transport to lysosomes. Therefore, and although their early steps are differently governed, autophagy and endocytosis can converge at a pre-lysosomal step or at the lysosomes to form hybrid organelles called, respectively, amphisomes or amphilysosomes [17, 19]. 

Ca^2+^ is a second messenger that is involved in the regulation of several physiological cell functions, such as gene transcription, metabolism, secretion and apoptosis, and perturbations in its homeostasis have been implicated in various pathological processes, such as disorders of the nervous system, cardiac and vascular pathologies and *diabetes mellitus* [20, 21]. Insights from the last years have deciphered some mechanisms that link Ca^2+^ with signalling and trafficking steps related with autophagy and endocytosis, but several details still remain unknown. Here we will review, consecutively, the role of Ca^2+^ in the regulation of: i) autophagy, ii) endocytosis, and iii) their final convergence into lysosomes for the degradation of the material taken up by these two processes. 

## 1 INVOLVEMENT OF CA^2+^ IN THE REGULATION OF AUTOPHAGY

### 1.1 Cytosolic Ca^2+^ Signaling in Autophagy

**Fig. (2):**
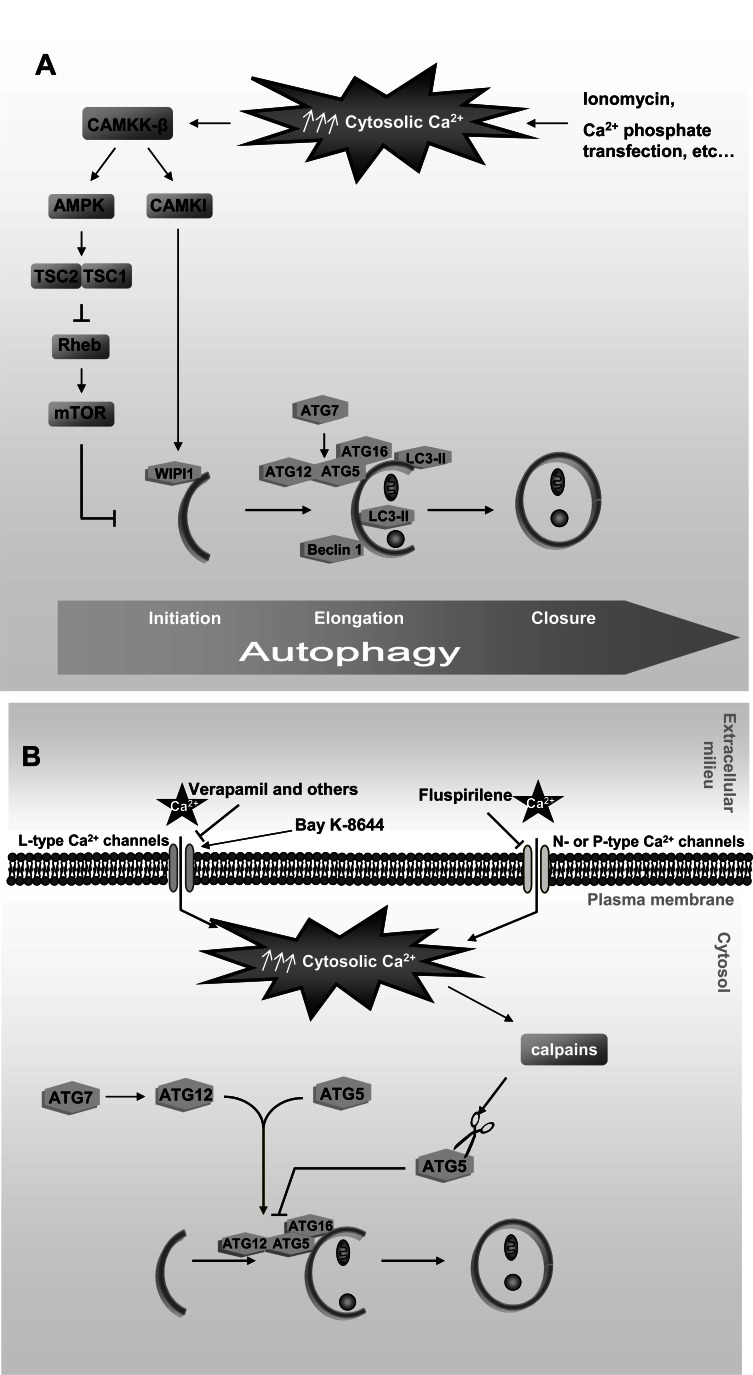
Cytosolic Ca^2+^ effects on autophagy. **A**. Cytosolic Ca^2+^ induces autophagy in non-excitable cells: Rise of cytosolic Ca^2+^ produced by different drugs and Ca^2+^ phosphate-mediated transient transfections activates the CAMKK-β-AMPK-mTOR and CAMKK-β-CAMKI signalling pathways that induce autophagy through various protein targets implicated in this process. **B**. Cytosolic Ca^2+^ inhibits autophagy in excitable cells: Antagonists of L-, N- or P-type Ca^2+^ channels (verapamil, fluspirilene etc…), and an agonist of L-type Ca^2+^ channels (Bay K-8644) modify cytosolic Ca^2+^ levels and consequently affect the activity of the Ca^2+^-dependent proteases calpains, including their *ATG5* cleavage that inhibits autophagy. See text for further details.

Direct evidence that cytosolic Ca^2+^ signaling activates autophagy was provided in a study performed in MCF-7, NIH3T3 and HeLa cells, where increasing cytosolic Ca^2+^ levels with pharmacological agents such as ionomycin induced autophagy in a Beclin 1- and ATG7-dependent manner [22] (see Fig. **2A**). Autophagy was activated by a signaling pathway, involving Ca^2+^/calmodulin-dependent kinase kinase-beta (CAMKK-β) and AMP-activated protein kinase (AMPK), which inhibits the serine-threonine kinase mammalian target of rapamycin (mTOR). This inhibition of mTOR occurs *via* the GTPase activating protein Tuberous Sclerosis Complex (TSC1/2) and its substrate, the Ras-family GTP binding protein Rheb that directly regulates the activity of mTOR [23]. This was also confirmed in HEK293 cells transfected with amyloid-β and using resveratrol, a naturally existing polyphenol that increases cytosolic Ca^2+^. Under these conditions, the CAMKK-β-AMPK signalling pathway becomes activated and inhibits mTOR, leading to the autophagic degradation of amyloid-β [24]. Moreover, autophagy activation by resveratrol has been reported to occur in MCF-7 cells by a non conventional mechanism independent from canonical Beclin 1 [25].

However, it has been reported that Ca^2+ ^can also induce autophagy *via* WIPI1 by an alternative pathway downstream of CAMKK-β that activates Ca^2+^/calmodulin-dependent protein kinase I (CAMKI) and bypasses AMPK [26]. Further support for the involvement of cytosolic Ca^2+^ in the induction of autophagy was derived from transfection experiments with calcium-phosphate precipitates in which it was observed that these precipitates activate autophagy in a Beclin 1- and ATG5-dependent way [27].

However, other results are in conflict with those described above, since they support an inhibitory effect of cytosolic Ca^2+^ on autophagy (see Fig. **2B**). Thus, using Ca^2+^ channel antagonists, such as verapamil, which inhibit a family of Ca^2+^-activated cysteine proteases, the calpains, autophagy was activated by a pathway independent of mTOR [28], whereas Ca^2+^ channel agonists inhibit autophagy *via* the cleavage of ATG5 by calpains, which in turn decreases the formation of the ATG12-ATG5 conjugate that is indispensable for the formation of autophagosomes [29].

Therefore, whether rises in the cytosolic Ca^2+^ activate or inactivate autophagy is still a matter of discussion. Of note, studies supporting inactivation of autophagy by cytosolic Ca^2+^ are based on the modulation of voltage-dependent Ca^2+^ channels (L-, N- or P-type Ca^2+^ channels) that exist only in excitable cells [28, 29], whereas activation of autophagy by cytosolic Ca^2+^ has been reported in non-excitable cells [22, 26, 27]. Given that in excitable cells cytosolic Ca^2+^ is mainly provided from the extracellular space by voltage-activated channels, whereas in non-excitable cells it is mainly released from intracellular stores *via *second messengers (such as inositol 1,4,5-trisphosphate (IP_3_)) [26], it is possible that different Ca^2+^-sensor proteins in both groups of cells activate distinct signalling routes that lead to opposite autophagic responses. 

### 1.2 Regulation of Autophagy by ER-Derived Ca^2+^

Earlier studies demonstrated a role of Ca^2+^ storage within cell compartments in autophagy stimulation [30]. Since then, the importance of ER-derived Ca^2+ ^for the autophagic activity has been confirmed by several experimental evidences. The ER lumen constitutes both the main intracellular Ca^2+^ store and the major site in the secretory pathway for the proper folding of proteins, which is carried out by a group of chaperones, most of them Ca^2+^-dependent [31-33]. Therefore, disturbances in Ca^2+^ homeostasis inside the ER cause stress that compromises the functionality of this organelle and of the cell. 

#### 1.2.1 Autophagic Response to the Inhibition of ER Ca^2+^-ATPases by Thapsigargin 

The first direct evidence of a possible connection between Ca^2+^ efflux from the ER and autophagy came from the observation of an induction of autophagy by thapsigargin [34]. This compound hampers the Ca^2+^ transport into the ER through Ca^2+^-ATPase pumps, rendering this store depleted of Ca^2+^ and, subsequently, provokes ER stress [34, 35]. Several evidences indicate that Ca^2+^ rather than ER stress is important for the induction of autophagy by thapsigargin, since its effect is abolished by the potent cell permeant Ca^2+^ chelator BAPTA-AM (1,2-bis(o-aminophenoxy)ethane-*N,N,N*’*,N’*-tetraacetic acid (acetoxy methyl ester)) [22, 36]. In fact, thapsigargin causes ER stress only after prolonged treatments (reviewed in [37]), while autophagy activation is evident at short times. Moreover, thapsigargin is able to induce autophagy in cells deficient in the unfolded protein response [38] and other compounds that deplete Ca^2+^ from the ER induce autophagy without altering the unfolded protein response [39]. All these data support the contribution of ER-derived Ca^2+^ to the activation of autophagy independently of ER stress. 

**Fig. (3):**
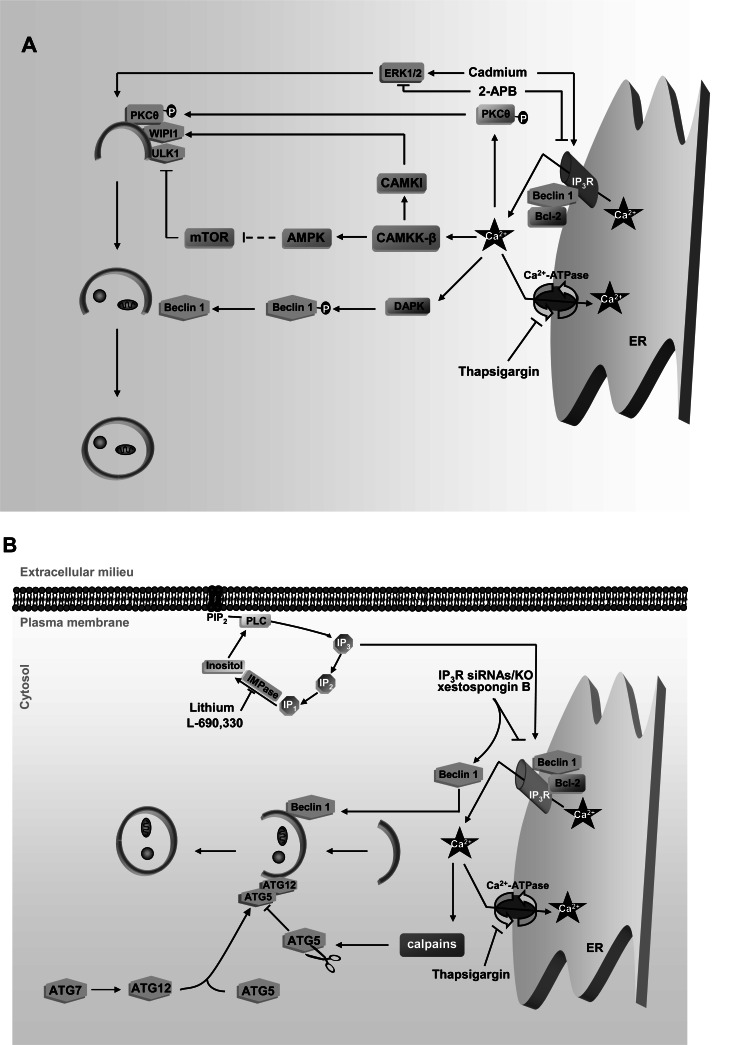
ER-derived Ca^2+^ effects on autophagy. **A**. Under starvation conditions, Ca^2+^ derived from the ER activates autophagy: ER depletion of Ca^2+^ by thapsigargin induces autophagy *via* the same signalling pathways from fig. 2A, and by a Ca^2+^-dependent phosphorylation of PKCθ that directs this kinase to autophagosomes. Ca^2+^ release from the ER through the IP_3_R is inhibited with 2-APB and induced with Cadmium and this inhibits and activates, respectively, autophagy *via* ERK1/2 signalling. Ca^2+^-dependent phosphorylation of Beclin 1 by DAPK also induces autophagy. **B**. Under full nutrient conditions, Ca^2+^ derived from the ER restrains autophagy: Thapsigargin inhibits autophagy *via* ATG5 cleavage by calpains. As regards IP_3_R function, inhibitors of inositol monophosphatases (IMPase), such as Lithium and L-690,330, which prevent IP_3_ generation and, hence, Ca^2+^ release through IP_3_R, induce autophagy. Also the inhibition of IP_3_R function with xestospongin B and knockdown/knockout of IP_3_R dissociates Beclin 1 from Bcl-2-IP_3_R complex and stimulates autophagy. IP: inositol 4 monophosphate; IP_2_: inositol 4,5 bisphosphate. See text for further details.

The Ca^2+^-dependent activation of autophagy by thapsigargin has been reported to occur in simple eukaryotes such as *Dictyostelium* [40], as well as in a wide range of mammalian cells (lymphocytes, hepatocytes and fibroblasts are some examples) [22, 36, 38, 41]. In *Dictyostelium* ATG1 is shown to be required [40], whereas in mammalian cells this Ca^2+^-dependent autophagy activation has been described to occur either via CAMKK-b-AMPK-mTOR signalling [22] that activates the mammalian homologue of ATG1, ULK1 (according to [42] and our unpublished results). Other possibilities for this autophagy activation include the participation of CAMKK-β-CAMKI [36, 41] or a Ca^2+^-dependent phosphorylation of PKCθ that recruits this PKC isoform to the autophagic vesicles [38] (see Fig. 3A). 

However, other studies have shown the opposite effect of thapsigargin [28, 30, 43, 44], and, as mentioned before, one of these studies ascribed this inhibition of autophagy to the Ca^2+^-dependent activation of calpains [28]. It seems that, in general and in accordance with what it was indicated in the previous section, in excitable cells autophagy is inhibited by thapsigargin, suggesting a negative role of ER-derived Ca^2+^ and hence of the Ca^2+^ supplied to the cytosol in this process. However, examples of non-excitable cells where autophagy is inhibited [28, 30] or activated [22, 38, 41, 45] are also observed. Given the diversity of the experimental conditions employed (0.01 to 5 μM of thapsigargin, for 15 min to 24 h), these differences could be due to side effects unrelated with the ER-derived Ca^2+^, since, for example, the use of BAPTA-AM in some of these studies does not rule out the involvement of Ca^2+^ present in other organelles. In fact, thapsigargin treatments at high concentrations and/or during prolonged times inhibit for example Ca^2+^-ATPase pumps at the Golgi complex [46]. Therefore, whether the Ca^2+^ released by thapsigargin from the ER activates or inhibits autophagy in non-excitable cells is still under debate.

Apart from Ca^2+^-ATPase pumps that control Ca^2+^ entry to the ER lumen, Ca^2+^ homeostasis in this organelle is also affected by Ca^2+^ release through the IP_3_ receptor (IP_3_R), an aspect that we discuss below.

#### 1.2.2 Regulation of Autophagy by IP_3_R-Dependent Ca^2+^ Release from the ER 

Efflux of Ca^2+ ^from the ER is mainly regulated by interaction of the second messenger IP_3_ with IP_3_R, resulting in the formation of a Ca^2+^ release channel at the ER [47]. IP_3_ is generated through the cleavage of phosphatidylinositol 4, 5-bisphosphate (PIP_2_) by phospholipase C (PLC), which can be activated by inositol recycled from inositol monophosphate by dephosphorylation [48]. Inhibitors of this inositol monophosphatase, such as Lithium, induce autophagy, suggesting a negative role of IP_3_ in the regulation of autophagy (see Fig. **3B**) [49, 50]. In accordance with this observation, various reports suggest that Ca^2+^ release through IP_3_R prevents autophagy, since inhibitors of this receptor, such as xestospongin B or dexamethasone, or the knockdown/knockout of all three IP_3_R isoforms induce autophagy [50-53]. This negative effect on autophagy of the Ca^2+^ released to the cytosol through IP_3_R appears to be only relevant under nutrient rich conditions, because in this situation, but not under starvation [51], the knockout of the three IP_3_R isoforms decreases mTOR activity and results in an increase of basal autophagy [52]. 

Moreover, this channel has been associated with two autophagy-related proteins, Bcl-2 and Beclin 1, which interact with IP_3_R forming a complex. Although Bcl-2 is not necessary for the *in vitro* binding of Beclin 1 to IP_3_R, it is indispensable for the complex formation in a cellular context and under full nutrient conditions [54]. However, starvation releases Beclin 1 from the complex with IP_3_R/Bcl-2 [54, 55] and this dissociation, which is a basic condition to activate autophagy, occurs when Beclin 1 is phosphorylated by the death-associated protein kinase (DAPK) [56]. Of note, interactors of Beclin 1, such as Bcl-XL and the nutrient deprivation factor NaF-1, are also part of this complex and are released from Beclin 1 and IP_3_R under starvation conditions [57-60]. Also, inhibition of IP_3_R by its knockdown or by xestospongin B disrupts the complex and leads to autophagy activation [50, 55]. Thus, IP_3_R probably acts as a scaffold to recruit proteins of the autophagic machinery under nutrient rich conditions.

As for the role of these autophagy-related proteins in IP_3_R function as a Ca^2+^ channel, it also seems to be dependent on the nutritional state of the cell, at least for the autophagy inducer Beclin 1. Under full nutrient conditions this protein does not affect Ca^2+^ release through IP_3_R [55], whereas under starvation Beclin 1 enhances the release of Ca^2+^ from the ER by IP_3_R in response to IP_3_ [54]. Moreover, Bcl-2, which inhibits autophagy by recruiting Beclin 1 to IP_3_R, reduces Ca^2+^ release through IP_3_R by a still unknown mechanism [61-64]. 

In conclusion, the impact of Ca^2+^ discharge from the ER through IP_3_R on autophagy appears to depend on two factors: the nutritional state of the cell and the scaffold properties of this channel to recruit autophagy-related proteins. Under full nutrient conditions, IP_3_R sequesters proteins essential for autophagy activation that do not affect Ca^2+^ release through this channel, whereas under starvation conditions these proteins are liberated and this increases both autophagy and Ca^2+^ release.

 Other drugs that increase (Cadmium) or inhibit (2-aminoethoxydiphenyl borate) Ca^2+^ efflux from the ER *via* IP_3_R, produce a similar effect (activation or inhibition, respectively) on autophagy *via *extracellular signalling-regulated kinase (ERK1/2) [65] (see Fig. **3A**). However, these chemicals are not necessarily specific for IP_3_R. For example, 2-aminoethoxydiphenyl borate is not a selective inhibitor of IP_3_R, because it also alters the activity of store-operated Ca^2+^ channels and Endoplasmic Reticulum Ca^2+^-ATPase (SERCA) pumps at the plasma membrane [66, 67] and activates mTOR and AMPK in a CAMKK-β independent manner (our unpublished results). Thus, probably the effect of these drugs on autophagy may not be exclusively due to the Ca^2+^ derived from the ER through IP_3_R. 

Overall, Ca^2+^ release from the ER through this channel appears to induce autophagy in starved cells, but to inhibit it under full nutrient conditions. As all these studies have been performed in non-excitable cells, this conclusion, at least under starvation conditions, is in agreement with the studies that proposed a role of cytosolic Ca^2+^ inducing autophagy in these cells.

### 1.3 Mitochondrial Link Between ER Derived Ca^2+^ and Autophagy

**Fig. (4):**
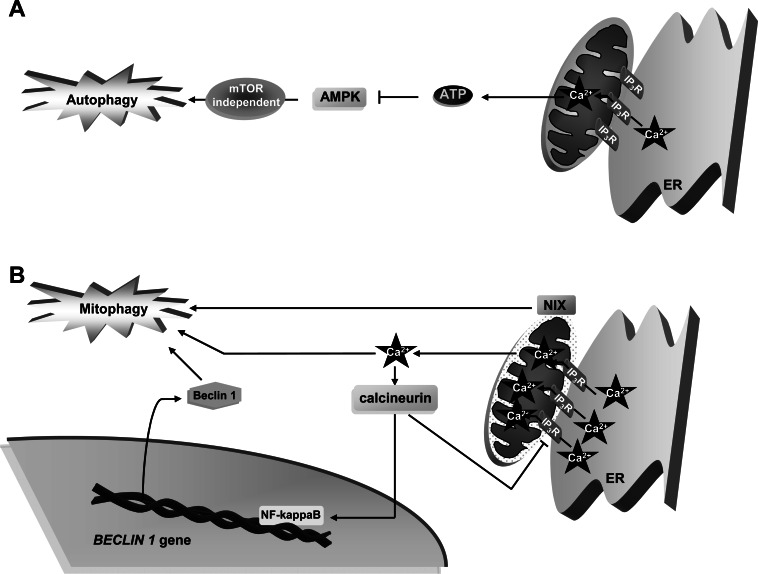
Effects of mitochondrial Ca^2+^ on autophagy. **A**. Mitochondrial Ca^2+^ inhibits autophagy: A moderate transfer of Ca^2+^ from the ER to mitochondria through IP_3_R, triggers ATP production that subsequently inactivates AMPK-dependent autophagy. **B**. Under stress, mitochondrial Ca^2+^ can activate autophagy: Mitochondria overloaded with Ca^2+^ are permeabilized and damaged. This promotes mitophagy and also activates calcineurin, which enhances NF-KappaB-mediated transcription of *Beclin 1.* Also, NIX buried in the outer mitochondrial membrane induces Ca^2+^ transfer from the ER to mitochondria and activates mitophagy. See text for further details.

IP_3_R is also found at ER-mitochondrial contact sites, since these two organelles are often found in close connection [50]. Thus, a blockage in Ca^2+^ release from the ER also alters Ca^2+^ homeostasis in mitochondria. The close proximity of ER and mitochondria is essential for an efficient transport of Ca^2+^ from the ER to mitochondria and the subsequent activation of Ca^2+^-dependent mitochondrial enzymes that participate in ATP production, such as pyruvate dehydrogenase (PDH), two enzymes of the Krebs cycle (isocitrate dehydrogenase and ketoglutarate dehydrogenase), and the F_1_F_0_ ATPase. Activation of PDH occurs by its dephosphorylation produced by the Ca^2+^-dependent stimulation of the PDH phosphatase (PDP). Although some cells, such as hepatocytes, express a PDP isoform whose activity is Ca^2+^-independent [68], the Ca^2+^-dependent activation of PDH by PDP seems to be a key step in many cells to supply them with NADH and ATP [69-72]. Thus, in HEK-293 cells that express PDP with Ca^2+^-dependent activity, when a moderate extent of Ca^2+^ (in the low micromolar range) is delivered to mitochondria, ATP increases, AMPK is inhibited and this restrains autophagy by an mTOR-independent signalling pathway [51] (see Fig. **4A**). 

On the contrary, under situations that may induce cell death, such as oxidative stress, a massive entry of Ca^2+^ (in the millimolar range) into mitochondria occurs as a consequence of its depolarization. This provokes the disruption of the integrity of the mitochondrial outer membrane and a rise in mitochondrial permeability [73, 74]. In most cells, these stress events provoke a specific autophagy (called mitophagy), which selectively degrades damaged mitochondria to preserve a healthy mitochondrial pool [75, 76] (see Fig. **4B**). 

Indirect links between mitochondrial Ca^2+^ overload and autophagy are provided by some proteins. The proapoptotic proteins Bcl-2 and adenovirus E1B 19-kDa-interacting protein 3 (BNIP3) and BNIP3-like, also known as NIX, participate in mitophagy induction in various cell types, including tumors, and localize on the outer mitochondrial membrane [77]. As NIX has been reported to trigger Ca^2+^ transfer from the ER to mitochondria in cardiac cells under stress conditions that may induce cell death [78], it is possible that this BNIP3-like protein uses this action to activate autophagy. However, further experiments are needed to confirm whether autophagy induction by these two proteins is due to an effect of NIX on mitochondrial Ca^2+^ overload and to generalize these observations to other cell types. 

Moreover, permeabilization of mitochondrial membranes under Ca^2+^ overload inside this organelle activates the cytosolic Ca^2+^-dependent phosphatase calcineurin [79], which further promotes autophagy (see Fig. **4B**) by dephosphorylation and inhibition of IP_3_R, constituting in this way a negative feedback to control Ca^2+^ release and to preserve mitochondrial homeostasis [65, 80]. Since calcineurin has been reported to be essential for the activation of NF-kappaB [81], a nuclear factor that, among other effects, enhances the transcription of Beclin 1 and induces autophagy [82], it is possible that this effect also contributes to the activation of autophagy observed in the mitochondrial Ca^2+^-mediated activation of calcineurin (see Fig. **3B**). 

In summary, mitochondrial Ca^2+^ regulates autophagy in two opposite ways. Moderate Ca^2+^ levels provided from the ER within mitochondria produce ATP that represses autophagy *via* inhibition of AMPK. On the other hand, when cells run into stress conditions, an excessive mitochondrial Ca^2+^ upload occurs that activates mitophagy by mechanisms involving pro-apoptotic proteins and probably calcineurin. 

Taken together the different Ca^2+^ stores in non-excitable cells, it seems that this cation and its sensor proteins in the cytosol induce autophagy when cells encounter conditions that require this process. Ca^2+^ release from the ER and mitochondria to the cytosol activates autophagy under stress conditions, whereas in healthy state, the storage of this cation inside these two organelles maintains low levels of autophagy. Thus, Ca^2+^ seems to participate in the adaptation of the autophagic level of the cells to their physiological state. As for excitable cells, although less attention has been paid to the Ca^2+^ impact on their autophagy, cytosolic Ca^2+^ seems to have the opposite effect on autophagy, probably because, as pointed above, the characteristics of the Ca^2+^-sensor proteins implicated in autophagy in these cells are different from the corresponding proteins in non-excitable cells.

## 2 INVOLVEMENT OF CA^2+^ IN ENDOCYTOSIS

**Fig. (5):**
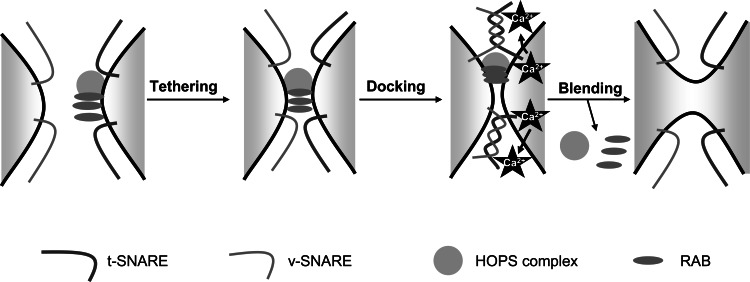
Main steps in the fusion of endocytic vesicles. First, the HOPs complex, RABs and other proteins are recruited to the target vesicle in order to allow tethering with the other vesicle. Subsequently, v- and t-SNAREs interact to allow the appropriate docking of the two opposite membranes. Ca^2+^ release from the target vesicle occurs at this stage to facilitate the blending of the two membranes. See text for further details.

During endocytosis, Ca^2+^ appears to be relevant in fusion/fission events [83, 84]. There are two different types of fusion: homotypic (early endosomes) and heterotypic (late endosomes-lysosomes), and their basic steps comprise: tethering, docking and, finally, blending of the membrane bilayers. Tethering starts with the binding of a complex of proteins including RABs and HOPS to the target membrane. Subsequently, membrane docking is promoted by the phosphoinositide (PIP)-dependent association of soluble NSF attachment protein receptors (SNAREs) to the two opposite membranes (v- for vesicle and t- for target) that finally culminate their fusion (Fig. **5**) [85-88]. In spite of their differences, fission and fusion events share several biochemical similarities and, for instance, RAB proteins and PIPs regulate both processes [89]. After docking, a release of luminal Ca^2+^ from endolysosomal compartments is thought to trigger fusion/fission events near Ca^2+^ release sites [83, 84, 90, 91]. This concept was evidenced for the first time using the intracellular Ca^2+^ chelators BAPTA and ethylene glycol tetraacetic** (**EGTA). In membrane fusion assays, BAPTA but not EGTA inhibits the fusion of late endosomes with early endosomes [92], lysosomes [93] or yeast vacuoles [84]. As at their maximal concentrations (10 mM) BAPTA binds Ca^2+^ in less time (0.3 μs) than EGTA (1.2 ms) [94], and since the Ca^2+^ diffusion rate in the cytosol is 20 nm/ms [95], this selective inhibition leads to postulate that the Ca^2+^ release source is situated at 20 nm or less from the site where fusion occurs, a reasonable distance to consider the lumen of vesicles committed to a fusion event as the source of this Ca^2+^. In fact, the depletion of luminal Ca^2+^ from these vesicles has the same effect on their fusion than BAPTA [93, 96].

It is believed that specific endolysosomal Ca^2+^-sensors transduce these Ca^2+^ signalling into a fusion response. The best studied sensor is calmodulin, which has been shown to be crucial in homotypic [92, 97] and heterotypic [84] fusions. Ca^2+^ binding to calmodulin leads to interactions between this protein and specific targets, such as calmodulin-dependent kinase II (CAMKII) [97] or a complex formed by early endosome antigen 1 (EEA1) [92] and the SNARE protein SYNTAXIN 13 [98], to promote early endosome fusions. Moreover, calmodulin has the ability to dislocate EEA1 from early endosomal membranes [92]. Thus, Ca^2+^/calmodulin may not only play the role of recruiting fusion effectors to early endosomes, but it can also recycle tethering molecules such as EEA1. 

Apoptosis-linked gene-2 (ALG-2), has been also proposed as a Ca^2+^-sensor for later fusion events in the endolysosomal system through its Ca^2+^-dependent interaction with the transient receptor potential cation channel, mucolipin subfamily 1 (TRPML1) [99], a putative endolysosomal ion channel involved in the transport of Ca^2+^ and other ions from the lysosomal lumen to the cytosol [100-102]. Since the release of Ca^2+^ from the lumen of vesicles is essential for their fusion, this channel may provide Ca^2+^ from endolysosomes for their fusion with endosomes and autophagic vacuoles [100, 103-106], hence the importance of lysosomal Ca^2+^ in these processes that we will discuss in the following section.

## 3 ROLE OF ENDOLYSOSOMAL CA^2+^ IN AUTOPHAGY AND ENDOCYTOSIS 

Fusion of autophagosomes and endosomes with lysosomes to deliver their respective cargo constitutes a late step in autophagy and endocytosis. Both groups of fusions share certain features, such as the involvement of RAB7 and the AAA ATPase SKD (Vacuolar protein sorting 4/suppressor of K^+^ transport growth defect 1) in their regulation [107, 108]. Although in comparison to the early stages of autophagy and endocytosis these late steps remain poorly understood, it is known that Ca^2+^ is a key player [83, 84]. Here below, we will review recent advances focused on the involvement of Ca^2+^ derived from lysosomes in the fusion of these organelles with autophagosomes and endosomes. 

### 3.1 Endolysosomal Ca^2+^ Channels

The best characterized Ca^2+^ channels present in lysosomal and late endosomal membranes are TRPMLs [100, 101, 106, 109]. Three isoforms (1, 2 and 3) have been identified in database searches [109] and mutations in the gene encoding TRPML1 provoke type IV mucolipidosis [110], a lysosomal storage disease. Although TRPML1 showed Ca^2+^-related features in endolysosomal compartments, such as Ca^2+^ permeability [101, 102], its consideration as a reliable Ca^2+^ channel is still under debate [111-113]. However, TRPML1, and TRPML2 as well, have been reported to heteromultimerize with TRPML3, which is the most accepted isoform to function as a Ca^2+^ channel [102, 114-116], and to control its lysosomal localization [117]. 

While there are no experimental evidences for a direct involvement of the two other isoforms in autophagy, recent data have shown that TRPML3 is localized on autophagosomal membrance, where it induces autophagy under stress conditions [105, 118], and also at the plasma membrane and early endosomal membranes, where it inhibits endocytosis [118, 119]. 

Two other candidates to function as lysosomal/endosomal Ca^2+^ release channels have recently emerged: Transient receptor potential cation channel, subfamily M, member 2 (TRPM2) and two-pore channels (TPCs). TRPM2, whose expression is restricted to specific cells, like pancreatic β cells, is mainly expressed at the plasma membrane, but it has been also localized on lysosomes, where it has been proposed to regulate luminal Ca^2+^ release [120]. TPC1 and TPC2 appear to be exclusively localized on early/late endosomes and lysosomes, respectively [121-123]. Both TRPM2 and TPCs are reported to be regulated by NAADP, a well-known endogenous second messenger that releases Ca^2+^ from acidic compartments [121, 124, 125].

Although NAADP-regulated TRPM/TPCs channels can release Ca^2+^ from endolysosomal compartments, knowledge on their specific role in autophagy and endocytosis remains rudimentary. In this regard, it has been suggested that Ca^2+^ release through NAADP-sensitive channels contributes, at least, to fusions between lysosomes and endosomes, since these channels are localized on these organelles [121-123]. 

Lysosomal Ca^2+^ is also regulated by pH. In fact, disruption of lysosomal pH by lysosomotropic agents, like bafilomycin A1, chloroquine diphosphate or nigericin, prevents Ca^2+^ storage in the lysosomal lumen and arrests the fusion of lysosomes with autophagosomes [126]. Therefore, an acidic pH is crucial to maintain high levels of Ca^2+^ in the lysosomal lumen, a requirement to induce fusions between lysosomes and endosomes or autophagosomes upon Ca^2+^ release from the lumen of these vesicles. In accordance with this concept, an *in vitro* study with isolated autophagosomes and lysosomes revealed that fusion between both organelles requires a minimum of 250 μM of calcium chloride [127].

### 3.2 Ca^2+^-Dependent Effectors of Endolysosomal Fusions

Another physiological feature of Ca^2+^ that is relevant in autophagy and endocytosis consists on its ability to promote the fusion of vesicles by inducing local segregations of specific lipids such as phosphatidic acid [128, 129]. Several *in vivo *and *in vitro* data support that these lipid domains are stabilized by proteins that bind to the membranes [130-132]. The best studied of these proteins belong to the SNARE machinery. First, this protein complex triggers docking of vesicles, which provokes a quick luminal Ca^2+^ release. Subsequently, Ca^2+^-binding proteins (that we will discuss below: see Table **1**) are activated, probably organizing a scaffold upon the membranes that initiates the fusion processes. Finally, after dissipation of the Ca^2+^ gradient, these proteins remain activated until fusion is accomplished [90]. 

A peculiar protein from the SNARE complex is Hepatocyte responsive serum phosphoprotein (HRS), a Ca^2+^-sensitive protein associated to early endosomes. When bound to a still undefined SNARE protein on membranes of early endosomes, HRS prevents homotypic membrane fusions, thus negatively regulating the fusogenic function of SNAREs [133]. Ca^2+^ release from the endosomal lumen dissociates HRS from the SNARE complex and abolishes this effect, enabling in this way endocytic fusion [134]. 

On the other hand, this protein has been also shown to partially colocalize with autophagosomes and to promote their maturation [135]. Somewhat related to these findings, a Ca^2+^-binding protein, annexin A5, has been shown to be recruited to lysosomal membranes in a Ca^2+^ dependent way, to induce autophagosome fusion with lysosomes and to inhibit endocytosis [136, 137]. The similarity between the roles on autophagy and endocytosis of HRS and annexin A5, together with the TRPML3 channel reported in the previous section (that also promotes autophagy and inhibits endocytosis), suggests that Ca^2+^ release from the autophagic/ endolysosomal vesicles may control the role of these proteins in autophagy and endocytosis. 

Calmodulin has been also proposed to be a Ca^2+^-sensor of SNAREs. The first evidences of this role were obtained in yeast, where calmodulin was identified within a protein complex involved in homotypic vacuole fusion [93, 138]. In mammalian cells, an implication of calmodulin in homotypic and heterotypic fusions was also proposed [84, 92, 97, 139, 140]. 

Moreover, some members of the annexin family are associated with fusion events in the endolysosomal system. *In vitro *studies showed the requirement of annexin A1 in fusions between early endosomes in a Ca^2+^-dependent manner [141], whereas *in vivo *analysis attributed to annexins A2, A5 and A6 the abilities to mediate the fusions of early endosomes [142], autophagosomes/ lysosomes [136], and late endosomes/lysosomes, respectively [143]. 

Overall, Ca^2+^-dependent effectors of fusions between autophagosomes, endosomes and lysosomes belong to a wide range of subgroups such as SNAREs, EF-hand proteins and annexins, with some common characteristics, including the requirement of Ca^2+^ binding. However, the molecular mechanisms by which they control these events are still poorly understood.

## CONCLUSIONS

Growing evidences support that Ca^2+^ controls endocytosis and autophagy. Its effect on autophagy occurs both at the level of the signalling pathways that initiate it or, later, when autophagosomes fuse with endolysosomal compartments.

The effect of Ca^2+^ on autophagy depends on the cell type, since excitable and non-excitable cells exhibit opposite autophagic responses (inhibition or activation, respectively) to this cation. Although less attention has been paid to excitable cells, Ca^2+^ rise within them restrains autophagy and this effect is mainly due to the activation of calpains that cleave proteins essential for autophagy. To decide whether other Ca^2+^-sensor proteins, specific or not for these cells, are also involved in this effect requires further work that would help to better understand the autophagic behavior of these cells.

**Fig. (6):**
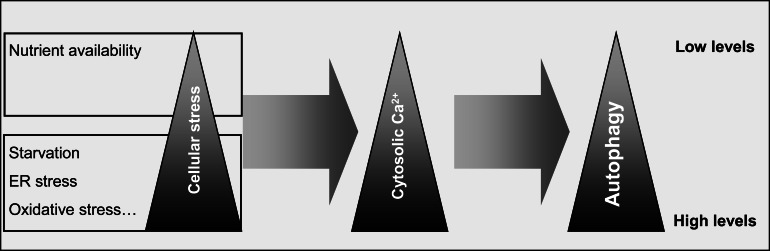
Possible relationships between nutrient availability, cell stress, cytosolic Ca^2+^ levels and autophagy. Starvation and an increased cell stress correlate with a high level of cytosolic Ca^2+^ generated from the ER and/or mitochondria and this induces autophagy. On the other hand, when nutrients are available to cells and no stress occurs, cytosolic Ca^2+^ remains at a low level and, consequently, basal autophagic activity is maintained.

In non-excitable cells, the effect of Ca^2+^ on autophagy depends on the nutritional state of the cells and, probably, on the Ca^2+^ levels within the cytosol. Under full nutrient conditions, Ca^2+^ levels in the cytosol are low and maintain a basal autophagy. Starvation and stress conditions induce a rise of cytosolic Ca^2+^ originated, respectively, from the ER and mitochondria overloaded with Ca^2+^. Subsequently, these conditions trigger autophagy *via* various pathways that depend on Ca^2+^-sensor proteins (Fig. **6**). Thus, in non-excitable cells, Ca^2+^ seems to play a protective role by adapting autophagic activity to extracellular conditions. Therefore, manipulation of intracellular Ca^2+^ levels in situations of defective autophagy may be useful to recuperate cellular homeostasis.

Concerning endocytosis, the traffic of endocytic vesicles is controlled by Ca^2+^ derived from their lumen and, subsequently, Ca^2+^-sensor proteins transduce this Ca^2+^ signalling into fusion events. 

Finally, the convergence of the autophagic and endocytic vesicles to lysosomes shares several features that depend on Ca^2+ ^originated from lysosomes/late endosomes and on proteins that are subsequently activated by this cation. However, the involvement of Ca^2+^ and its effects on sensor proteins in these final autophagic and endocytic stages remain poorly understood. Although various members of these proteins have been identified, further investigations are needed to identify new Ca^2+^ effectors and their role in the regulation of the different steps of autophagy and endocytosis.

## Figures and Tables

**Table 1.** Ca^2+^-Dependent Effectors Involved in the Fusions Between Lysosomes, Autophagosomes and/or Endosomes.

Ca^2+^-dependent effectors	Organelles participating in the fusion event	Molecular details of their role	References
ALG-2	Late endosomes and lysosomes	Interacts with TRPML1 channel	[99]
Annexin A1	Early endosomes	Requires Ca^2+^ to induce fusion *in vitro*	[141]
Annexin A2	Early endosomes	Mediates membrane interactions between early endosomes	[142]
Annexin A5	Autophagosomes and lysosomes	Translocates, under starvation, to lysosomes in a Ca^2+^-dependent way	[136]
Annexin A6	Late endosomes and lysosomes	Requires Ca^2+^ and calpains for fusion	[143]
Calmodulin	Late endosomes and lysosomes Early endosomes	Its binding to Ca^2+^ leads to interactions with specific targets	[84]
CAMKII	Early endosomes	Calmodulin target	[97]
EEA1	Early endosomes	Interacts with calmodulin and SYNTAXIN 13	[92]
HRS	Early endosomes Autophagosomes and lysosomes	Inhibits fusion when Ca^2+^ release abolishes its interaction with SNAREs	[134] [133] [98]
SYNTAXIN 13	Early endosomes	Interacts with Ca^2+^/calmodulin to promote early endosome fusions	[98]

## LIST OF ABBREVIATIONS

ALG-2: = Apoptosis-linked gene-2
AMPK: = AMP-activated protein kinase
ATGs: = Autophagy-related genes
BAPTA-AM: = 1,2-bis(o-aminophenoxy)ethane-*N,N,N*’*,N’*-tetraacetic acid (acetoxy methyl ester)
Bcl-2: = Protein B-cell lymphoma/leukemia 2
BNIP3: = Bcl-2 and adenovirus E1B 19-kDa-interacting protein 3
CAMK: = Ca^2+^/calmodulin-dependent protein kinase
CAMKK-β: = Ca^2+^/calmodulin-dependent kinase kinase-beta
DAPK: = Death-associated protein kinase
EEA1: = Early endosome antigen 1
EGTA: = *Ethylene glycol tetraacetic*
ER: = Endoplasmic reticulum
ERK1/2: = Extracellular signalling-regulated kinase
HRS: = Hepatocyte responsive serum phosphoprotein
IP_3_: = Inositol 1,4,5-trisphosphate
IP_3_R: = Inositol 1,4,5-trisphosphate receptor
PIP: = Phosphoinositide
mTOR: = Mammalian target of rapamycin
PDH: = Pyruvate dehydrogenase
PDP: = PDH phosphatase
SNARE: = Soluble NSF attachment protein receptors
TRPM2: = Transient receptor potential cation channel, subfamily M, member 2
TRPML: = Transient receptor potential cation channel, mucolipin subfamily
TPCs: = Two-pore channels
ULK1: = UNC-51 Like Kinase
